# The role of reactive oxygen species in tumor treatment

**DOI:** 10.1039/c9ra10539e

**Published:** 2020-02-24

**Authors:** Pengpeng Jia, Chenyu Dai, Penghui Cao, Dong Sun, Ruizhuo Ouyang, Yuqing Miao

**Affiliations:** Institute of Bismuth Science, University of Shanghai for Science and Technology Shanghai 200093 China ouyangrz@usst.edu.cn; School of Chemistry and Chemical Engineering, Henan Normal University Xinxiang 453007 China sundong2004@126.com

## Abstract

Reactive oxygen species (ROS) are by-products of aerobic metabolism and can also act as signaling molecules to participate in multiple regulation of biological and physiological processes. The occurrence, growth and metastasis of tumors, and even the apoptosis, necrosis and autophagy of tumor cells are all closely related to ROS. However, ROS levels in the body are usually maintained at a stable status. ROS produced by oxidative stress can cause damage to cell lipids, protein and DNA. In recent years, ROS have achieved satisfactory results on the treatment of tumors. Therefore, this review summarizes some research results of tumor treatments from the perspective of ROS in recent years, and analyzes how to achieve the mechanism of inhibition and treatment of tumors by ROS or how to affect the tumor microenvironment by influencing ROS. At the same time, the detection methods of ROS, problems encountered in the research process and solutions are also summarized. The purpose of this review is to provide a clearer understanding of the ROS role in tumor treatment, so that researchers might have more inspiration and thoughts for cancer prevention and treatment in the next stage.

## Abstract 2

This review provides a clear understanding of the ROS role in tumor treatment and some thoughts for potential cancer prevention.

## Graphical abstract

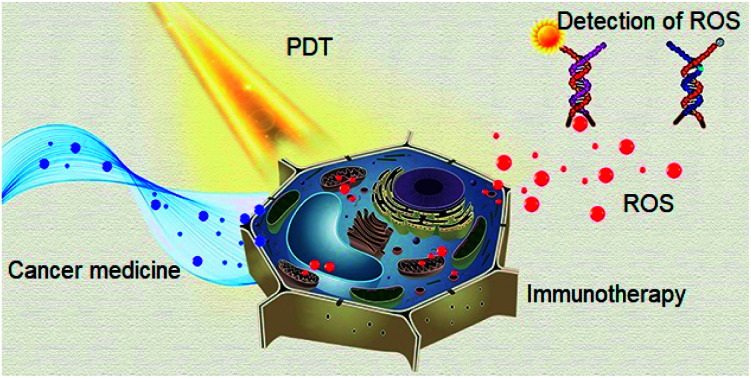

## 1. Introduction

**Fig. 1:**
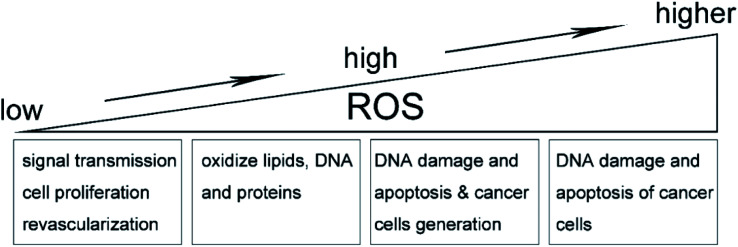
Effect of ROS on tumor microenvironment.

Reactive oxygen species (ROS) are a series of molecules including singlet oxygen (primary excited state), superoxide anion (single electron state), hydroxyl radical (three-electron state) and hydrogen peroxide (double-electron reduction state),^1^ which are generated through intracellular oxidation metabolism. It is worth noting that, at a low level, ROS can be involved in multiple regulation of biological and physiological processes as important signaling molecules. Intracellular ROS levels normally remain stable, because ROS are essentially strong oxidants that can participate in oxidative stress by raising intracellular levels to damage oxidize lipids, DNA, and proteins.^2,3^ On the other hand, in the tumor microenvironment, ROS in relatively low levels play an important role in signal transfer, cell proliferation and revascularization, and the gradual increase of ROS can also promote the cell proliferation, diffusion and metastasis of tumors. On the contrary, ROS in high levels will damage the DNA of cancer cells, resulting in apoptosis and tumor necrosis to some degree.^4–6^ Therefore, the antioxidant activity of relative magnitude will be generated to balance the high concentration of ROS, as shown in Fig. 1. In conclusion, the balance between intracellular oxidants (ROS) and antioxidants is very important. Anti-oxidants include enzymes (catalase, dismutase and peroxidase), nonenzymes (vitamin A, C and E) as well as nicotinamide adenine dinucleotide phosphate (NADPH) oxidase.^7^ Generally, during the oxidative phosphorylation of mitochondria in normal cells, the electron leakage from electron transfer chains and the reduction of oxygen molecules are the main sources of intracellular ROS.^8^ Most ROS can be generated by mitochondria and some enzymes, as summarized below.

### 1.1 ROS from mitochondria

For most cells, the respiratory chain of mitochondria is the main source to produce ROS. Since mitochondria are the “energy factories” of the body. Over 90% of the oxygen in the body is consumed by mitochondria to produce adenosine triphosphate (ATP) for various life activities. But, at the same time, ROS free radicals produced by mitochondrial oxygen consumption also accelerate the oxidation and damage of each cells, leading to disease or aging.^9^ Mitochondria are engaged in oxidative metabolism all the time, and there are a series of large multi-subunit protein complexes on the inner membrane of mitochondria. These complexes facilitate the flow of large numbers of electrons from substrates such as reductive coenzyme (nicotinamide adenine dinucleotide, NADH).^4^ The energy from the flow of electrons is used to facilitate proton gradient transport across the inner membrane of mitochondria, and which in turn promotes the formation of ATP. This method is used to generate ATP in an oxygen-dependent form, where oxygen molecules are reduced to a single-electron reduction product called superoxide anion (O_2_^−^).^10^ ROS produced from the mitochondrial respiration mainly refers to superoxide anions and their derivatives including HO_2_˙, H_2_O_2_, OH˙ and ^1^O_2_.

### 1.2 ROS from NADPH oxidase

In addition to mitochondria, NADPH oxidase is one of the main enzymes in the body that can promote ROS production. It is a multi-component enzyme containing membrane bound cytochrome b558 (p22^phox^ and gp91^phox^ heterodimer) and cytosolic regulatory proteins (p40^phox^, p47^phox^, p67^phox^ and RAC1).^11^ The subtypes belonging to NADPH oxidase are known as NOX oxidases in the presence of neutrophils. The main source of ROS has been proved to be the complex of flavin and heme proteins that transfer electrons from NADPH in the cytoplasm to molecular oxygen to produce the superoxide anion O_2_^−^.^4^ One of the subtypes of NADPH oxidase is NOX2, also known as gp91^phox^. NOX2 is mainly expressed in the endothelial, adventitial and phagocytic cells, and plays a physiological role in immune prevention and blood pressure regulation. Under the stimulation of neutrophil and cytoplasmic factors including p40^phox^, p47^phox^ and p67^phox^, RAC1 will rapidly supplement and combine with the fixed factors NOX2 and p22^phox^ on the cell membrane, producing a large number of phagocytic cells. The activated neutrophils eventually produce large amounts of ROS. At present, seven known homologues of NOX oxidase (NOX1, NOX2, NOX3, NOX4, NOX5, DUOX1 and DUOX2) can produce ROS in different degrees for some physiological functions such as immune defense and signal transmission.^12^

### 1.3 ROS from other enzymes

Besides the mitochondria and the subtypes of NADPH oxidase, there are also some other enzymes such as xanthine oxidase, nitric oxide synthase, cyclooxygenase, lipoxygenase and P450 cytochrome, which can produce ROS.^4^ In addition, endoplasmic reticulum and peroxisome can also produce some ROS. Previously, ROS were regarded as by-products of enzymes or as potential oxidants for Fenton reaction. For example, hydrogen peroxide combines with intracellular Fe^2+^ to oxidize carboxylic acid, alcohol or lipid into inorganic states.^13^ However, with the deepening of research, it has been found that ROS can also play a role in signal transmission as signaling molecules. For instance, hydrogen peroxide produced by the P450 cytochrome enzyme in the adrenal gland plays an important role in corticosteroid production by regulating an oxygen-dependent signaling pathway in the adrenal cortex.^14^

## 2. ROS and tumor

Cancer is one of the most serious diseases that human beings face and has seriously affected human health. Hence, the exploration of tumor prevention and treatment is also one of the focuses of current research. Many strategies have been developed to inhibit the generation, growth, spread and metastasis of tumors. Hypoxia, high pressure, acidity, vigorous metabolism and dense blood vessels are known as typical characteristics of tumor microenvironment. Because of the rapid growth rate, strong reproduction ability and high metabolism of tumor cells, their demand for nutrients is higher than that of normal cells, so the consumption of oxygen, glucose and other components is higher. On the other hand, the anaerobic respiration of tumor cells produces lactic acid through glycolysis, while the over-expressed carbonic anhydrase catalyzes CO_2_ and H_2_O to form carbonic acid, which is the main reason for the acidic microenvironment of tumor.^15^ A large number of oxidation reactions make the concentration of ROS free radicals in tumor higher than that in normal tissues. Therefore, ROS is closely related to various stages of tumor, and can indeed produce therapeutic effect to some extent on tumor. The treatment of tumors by ROS is well discussed from the perspectives of ROS and photodynamic therapy, ROS and cancer medicine, as well as ROS and immunotherapy.

### 2.1 ROS and photodynamic therapy

Photodynamic therapy (PDT) is a very effective interventional therapy for the treatment of tumors in recent years. During the PDT, the photosensitizer (PS) loaded inside nanomaterials converts surrounding oxygen into ROS through election transfer under the irradiation of a specific wavelength of light, leading to more ROS produced.^16^ The further increase in ROS level inside tumor microenvironment will break the balance of oxidation–antioxidation, inhibit the growth of tumor, damage the DNA and thus induce the apoptosis of cancer cells.^17^ Although PDT shows high efficiency on tumor therapy, there are three major bottle necks that puzzle the research progress, including the insufficient penetration depth of the external light source, the PS toxicity caused by non-specific targeting as well as the insufficient PDT efficiency caused by hypoxic hypoxia in tumor microenvironment.

In order to solve the insufficient PDT efficiency caused by the hypoxic hypoxia in tumor microenvironment, Z. Luo *et al.* designed a bionic artificial red blood cell loaded with oxygen carrier (hemoglobin) and PSs (indocyanine green, ICG).^18^ The nanosystem has the dual capability of stably and continuously delivering oxygen and acting as a fluorescent probe for real-time monitoring. Under the irradiation of near-infrared light, the loaded PS combined with the oxygen inside hemoglobin and produced a large amount of cytotoxic ROS that damaged tumor cells and nearby vascular systems. In addition, the multifunctional carbon-dot-decorated Ag/Au bimetallic nanoshells (CAANSs) was prepared as plasmonic catalytic nanoprobe, which acted like a smart nanozyme responding to the tumor microenvironment and killing the cancerous cells through cell apoptosis induced by the effective conversion from cellular H_2_O_2_ to cytotoxic O_2_^−^.^19^ With the aim to overcome the PS toxicity caused by non-specific targeting, B. Feng *et al.* synthesized a multifunctional prodrug nanosystem loaded with hexadecyl-oxaliplatin-trimethyleneamine (prodrug, HOT) and derivative of Chlorin e6 (acid-activatable PS, AC).^20^ After the nanosystem was modified with iRGD, a targeting ligand, it could function for tumor homing and penetration. AC will minimize the toxicity during the blood circulation, while its specific acid-response in orthotopic or metastatic tumor could enhance the fluorescence imaging and PDT. The synergistic effect of PDT and chemotherapy was well performed under the guide of acid-response fluorescence imaging.

**Fig. 2:**
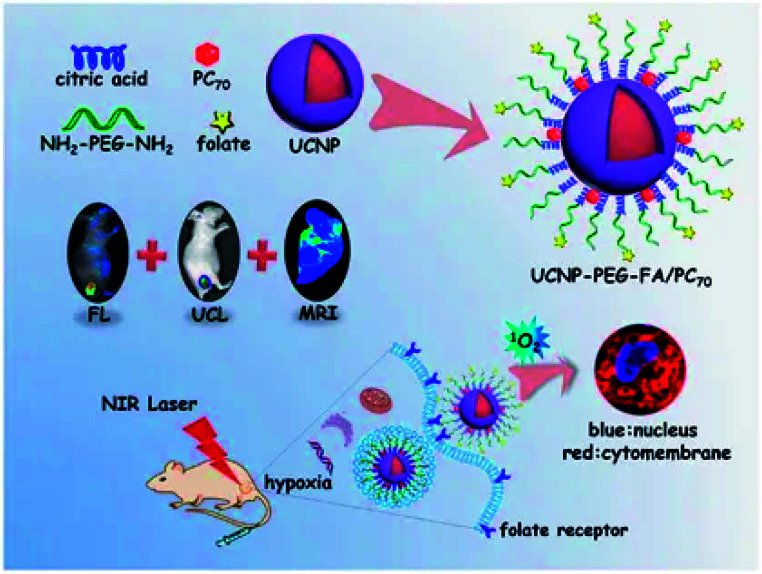
The production and function mechanism of UCNP-PEG-FA/PC_70_. This figure has been adapted for free from ref. 21 licensed under the Creative Commons Attribution 4.0 International License of Spring Nature (https://creativecommons.org/licenses/by/4.0/).

It is worth noting that novel multifunctional nanocomposites that could be excited by near-infrared light were developed.^21^ The new nanocomposites were based on frequency-increasing up-conversion nanomaterials (UCNPs) doped with lanthanide elements, and externally modified with porphyrin derivative-fullerene (PC_70_), where the large conjugated π bond in porphyrin-based structures make them exhibit excellent photophysical property and high singlet oxygen yield. The novel multifunctional nanocomposites were also used as fluorescence probes in pharmacokinetic due to the strong fluorescence property of porphyrin. PC_70_ has an extremely long triplet lifetime even under hypoxic conditions, enabling the generation of ^1^O_2_ under the excitation.^22^ It was then modified on the folate modified polyethylene glycol (PEG) surface to enhance active targeting and cycle time, as shown in Fig. 2. First of all, the lanthanide doped UCNPs could not only increase the conversion of natural anti-stokes frequency to produce cold light, but also have excellent light resistance and low background self-fluorescence. On the other hand, the conversion efficiency from near-infrared photons into UV-visible light was also very high.^23,24^ As a result, the as-designed UCNPs were capable for both multi-functional imaging and real-time monitoring. Also, the conversion of near-infrared light to UV-visible light could further stimulate PC_70_ to produce ^1^O_2_ in the hypoxic tumor microenvironment for the purpose of PDT. This method creatively avoided the lack of sufficient penetration depth of external light source, and also reduced the toxicity of PS through PEG modification.

**Fig. 3:**
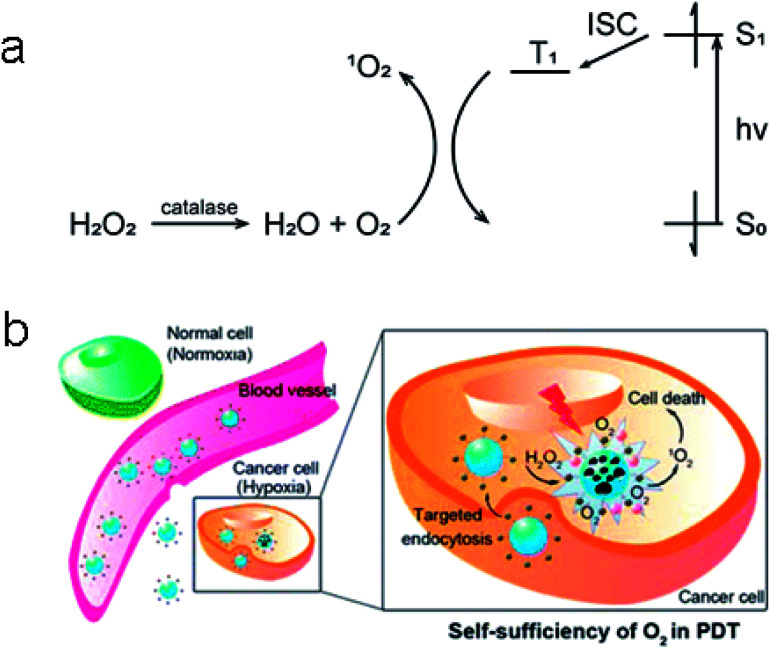
(a) Catalysis of H_2_O_2_ to O_2_ with catalase and conversion of O_2_ to ^1^O_2_ by MB (S_0_: singlet ground state; S_1_: lowest singlet excited state; T_1_: lowest triplet excited state; ISC: intersystem crossing). (b) The function mechanism of the synthesized nanoparticles. These figures have been adapted from ref. 25 with permission from American Chemical Society.

In addition, tumor cells are more sensitive to H_2_O_2_ than normal cells, too much or too little H_2_O_2_ will induce the apoptosis of tumor cells or even lead to the death of tumor cells. Based on this, H. Chen *et al.* designed and synthesized a H_2_O_2_-actived nanomaterial that can generate O_2_ and be used for PDT.^25^ The shell of the nanomaterial was a copolymer (poly(d,l-lactic-*co*-glycolic acid), PLGA) doped with black hole quencher-3 (BHQ-3), which could enhance the biocompatibility and biodegradability of this nanomaterial. As a PS, methylene blue (MB) would produce lots of ^1^O_2_ after being irritated with wavelength between 600–900 nm.^26^ And then, MB, together with catalase, would be incorporated into the core of the nanomaterial. Finally, a cyclic pentapeptide c(RGDfK) as a targeting ligand was modified outside the PLGA shell. Once the nanomaterial targeted into tumor cells, the abundant H_2_O_2_ in tumor cells would enter into the interior of nanomaterial and be catalyzed by catalase to produce O_2_ (Fig. 3). As a consequence, PLGA broke to release MB so that it could convert O_2_ to ^1^O_2_ under the laser irradiation, leading to efficient PDT.

At present, although PDT have been verified to perform with significant therapeutic effect, there are still three main problems needing to be addressed: how to further effectively increase the tissue penetration depth of the light source; how to further enhance the excitation efficiency of the photosensitizer and reduce the toxicity to normal cells; how to further improve the hypoxic state of tumor microenvironment so as to increase the ROS yield.

### 2.2 ROS and cancer medicine

**Fig. 4:**
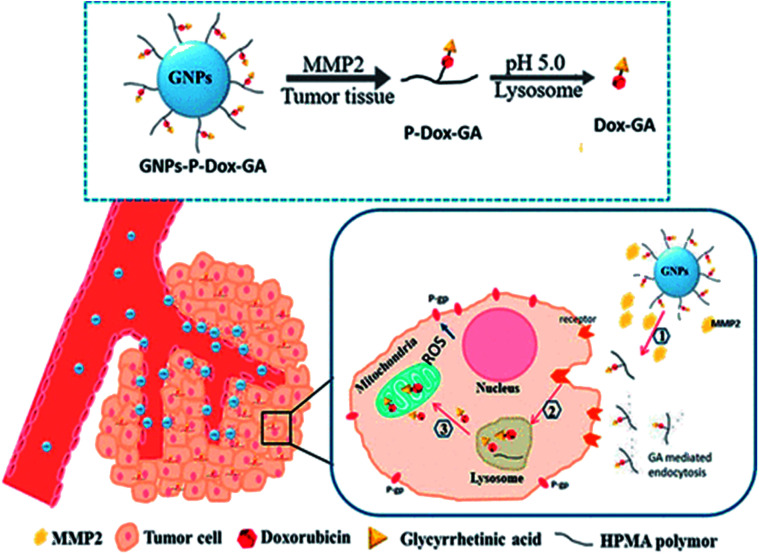
Mechanism illustration of the mitochondria targeting nanoparticle system (GNPs-P-Dox-GA). This figure has been adapted from ref. 28 with permission from American Chemical Society.

Most cells can buffer a certain amount of ROS through the reduction mechanism of the glutathione (GSH) redox system, but the long-term ROS in high level can cause cell damage.^27^ External factors such as radiation, air pollution and chemotherapeutic drugs can stimulate mitochondria to produce excessive ROS, induce the mitochondrial outer membrane pores to open, release calcium ions, and cytochrome C, and thus cause apoptosis. Using this mechanism to overcome the hepatocellular carcinoma multidrug resistance (MDR), Y. Liu *et al.* designed a mitochondrion targeting nanoparticle system (GNPs-P-Dox-GA), where doxorubicin (DOX) could accumulate in mitochondria by targeting mitochondria with glycyrrhetinic acid (GA), so as to generate a large amount of ROS and cause apoptosis (Fig. 4).^28^

Although the chemophotodynamic therapy has a significant synergistic effect on malignant tumors, currently available nanocarriers have limited capabilities in selective toxicity, drug release and tumor penetration. A poly ethylene glycol (PEG)–stearylamine (C18) conjugate (PTS) self-assembled with ROS-sensitive thioketal linker (TL), and was combined with the co-loaded doxorubicin (DOX) and photosensitive decolorant A (PhA) to enhance the local chemophotodynamic therapy.^29^ This new light-activated nanomicelles effectively delivered two drugs to enhance the chemophotodynamic therapy and minimize side effects.

### 2.3 ROS and immunotherapy

Adoptive immunotherapy (AI), a novel antitumor strategy, will be introduced here where the influence of ROS in AI or how does the therapy affect tumor metabolism through affecting ROS was described. AI generally refers to the transfer of donor lymphocytes into the recipient to enhance the cellular immunity of the recipient. Generally, AI can be divided into two categories: specificity – the transfer of a known antigen-sensitized lymphocytes to a receptor so that it is immune to the antigen; non-specificity – the transfer of normal, non-antigenic lymphocytes to a receptor to gain immunity to multiple antigens. By activating or amplifying tumor-specific or non-specific cells *in vitro*, AI can stimulate and repair the body's cellular immunity against diseases and infections, enhance the anti-tumor immunity of the tumor microenvironment, and thereby control and kill tumor cells. At present, T cell adoptive immunotherapy (ACT) is a clinically effective immunotherapy for tumor. The T cell used in ACT can be tumor-infiltrating lymphocytes from *in vitro* expansion; it can also be designed to express tumor-specific antigen T cell receptor (TCR) or a chimeric antigen receptor (CAR).^30^

**Fig. 5:**
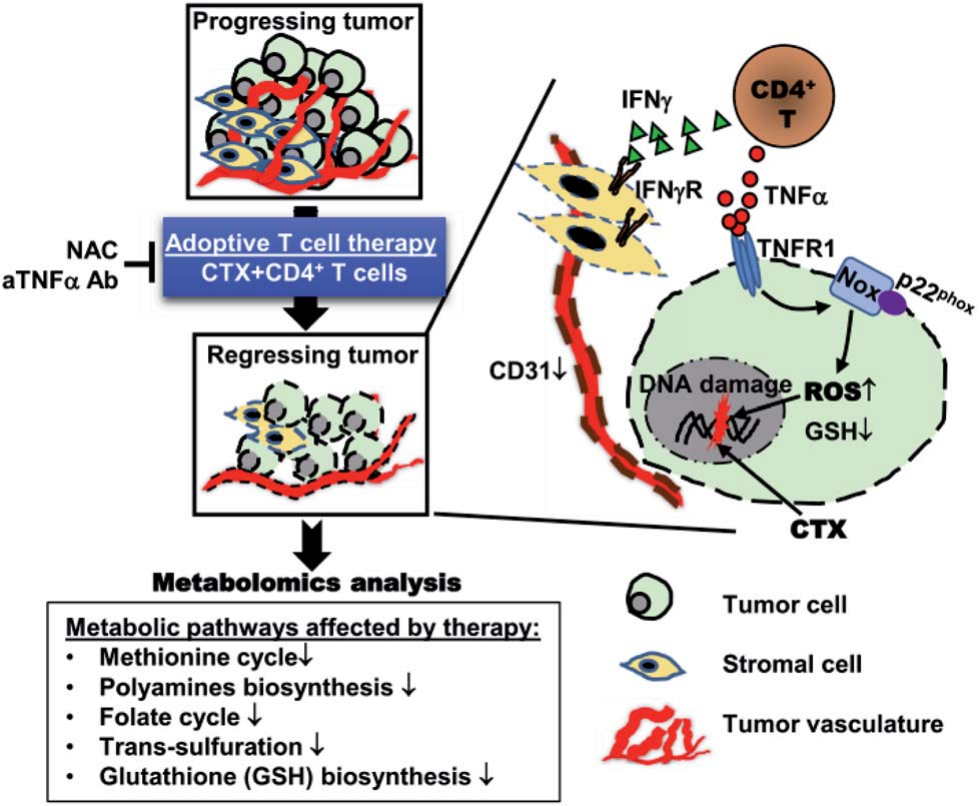
Illustration of the mechanism of CD4+ T cell-based adoptive immunotherapy. This figure has been adapted from ref. 30 with permission from Elsevier.

In view of the need for tumor cells to adapt to their high energy demands and uncontrolled growth, tumor therapy targeted at the metabolic pathways required for the survival and growth of tumor cells is also a promising idea.^31^ T. Habtetsion and his colleagues altered tumor metabolism through CD4 positive T cells, leading to the increase in the oxidative stress dependent tumor necrosis factor (TNF-) and tumor cell death, as shown in Fig. 5.^30^ Adoptive transfer (AT) of tumor specific CD4 positive T cells pretreated with cytoxan (CTX) was first proved to produce multifunctional CD4 positive effector cells. These cells produced inflammatory cytokines (such as TNF-α, interferon γ) and promoted the decay of vessel-intensive tumor. Second, it was also found that CTX + CD4 AT greatly destroyed the multiple metabolic ways of the tumor, and thus caused the generation deficiency of GSH, the main antioxidant inside cell. The tumor microenvironment-oxide antioxidant balance was broken by the weakened antioxidant capacity, resulting in the tumor cell DNA damage due to the accumulation of a large number of ROS. In addition, they demonstrated that TNF-α needs to be used in conjunction with chemotherapy to enhance ROS in tumor sites through mechanisms such as NADPH oxidase. From the perspective of tumor metabolism pathway, this research group changed tumor microenvironment oxidative stress to produce a large amount of ROS by changing tumor metabolism to produce a series of cytokines, thus killing tumor cells.

T cells are lymphoid stem cells in bone marrow that are distributed to the immune organs of the whole body through lymph and blood circulation after the differentiation and maturation of thymus. T cells not only need to be able to reach the target site, but also need to be able to survive there and develop immunity. Adoptive T cell therapy has achieved good clinical results, but the treatment effect of T cells on the tumor can be inhibited owing to the high inflammatory activity and the presence of a large number of ROS at the tumor site. To solve this problem, M. Ligtenberg and his colleagues remolded T cells to co-express catalase (CAT) and tumor-specific CAR to metabolize high level H_2_O_2_ so as to improve their antioxidant capacity.^32^ On the one hand, these novel T cells (CAR-CAT-T cells) can significantly increase the intracellular catalase level, and at the same time reduce the accumulation amount of ROS at the tumor site no matter in the ground state or in the activated state, thus reducing oxidative stress (oxidative stress occurs when the balance between ROS production and antioxidant function is broken). On the other hand, CAR-CAT-T cells can lyse tumor cells in antigen-specific ways under the oxidative stress induced by H_2_O_2_. Finally, CAR-induce a protective side effect, that is, the destruction of tumor cells by peripheral natural killer (NK) cells. This treatment strategy provides a way to maintain antitumor activity by adoptive transfer of immune cells (T cells) under hypoxia and oxidative stress.

Immunotherapy mainly uses immune T lymphocytes or natural killer (NK) cells to carry out immunotherapy on the receptor in an adoptive way. On the one hand, these immune cells have the ability to resist the invasion of individual inflammation and the formation of lesions. On the other hand, the “modified” immune cells can to some extent wake up the activity of the recipient's own immune factors to affect the homeostasis of tumor microenvironment (such as ROS, pH, *etc.*), thus inhibiting or killing the tumor cells. Tumor immunotherapy, which works by strengthening the body's own immune system and removing tumor cells from the body, is one of the hottest areas of immunotherapy, particularly the checkpoint therapy, by James Alison and Benjamin, the winners of the 2018 Nobel Prize in physiology or medicine. However, although some immunotherapy drugs have been used in clinical practice, this method mainly aims to remove the “shackles” of human immune cells, and enhance their ability to recognize surface factors of tumor cells, thus affecting tumor cells. Unavoidably, this approach can lead to an overactive autoimmune response that can cause serious adverse reactions and even life-threatening ones. Therefore, how to control more effectively and reliably and elucidate the mechanism of action is one of the main research points of immunotherapy in the next step.

## 3. Detection method

As a series of molecules that can be used to kill tumor cells, ROS have strong activity *in vivo*, but their life span is very short. ^1^O_2_ has an average life of about 2 μs, OH˙ about 200 μs, and O_2_^−^ about 5 s. Hence, how to detect the ROS produced by appropriate methods is also worth thinking about. Currently, the methods for ROS detection reported include election paramagnetic resonance (EPR),^33^ spectrophotometry,^34^ chemiluminescence and fluorescence analysis.^35,36^ Fluorescence analysis is widely used because fluorescence probe has good stability, cell membrane permeability and low cytotoxicity.^37,38^

The working principle of EPR is that the microwave energy resonates with atoms, ions or molecules of unpaired electrons under the action of a stable magnetic field.^33^ However, the direct detection of ROS in actual samples using EPR spectroscopy is often difficult owing to the instability of ROS. Moreover, the instruments to detect ROS are expensive and the analysis of results is complicated, so ERP is not effective in practical applications. Spectrophotometry cannot perform real-time analysis of ROS in cells and animals due to the poor sensitivity and is generally used for detection in solution only. The chemiluminescence method has excellent sensitivity and selectivity, but the high background light in animals greatly affects its reproducibility, and the actual testing effect is not satisfactory. The fluorescence analysis also suffers from the too short fluorescence and is susceptible to interference.

ROS can be successfully detected by some chemical probes. The ideal chemical probes can still detect the targets efficiently even at very low concentrations of ROS or non-radical products.^39^ Although the use of chemical probe is also different, ROS produced from different tissues, cells or even some intracellular organelles, are basically analyzed by fluorescence or cold light changes under the irradiation of light source with specific wavelength, through macroscopic observation (such as fluorescent microscope) or micro calculation (like flow cytometry). This part mainly summarizes the different detection probes toward ROS (H_2_O_2_, O_2_^−^, ^1^O_2_) in tumor therapy in recent years.

### 3.1 Detection of H_2_O_2_

**Fig. 6:**
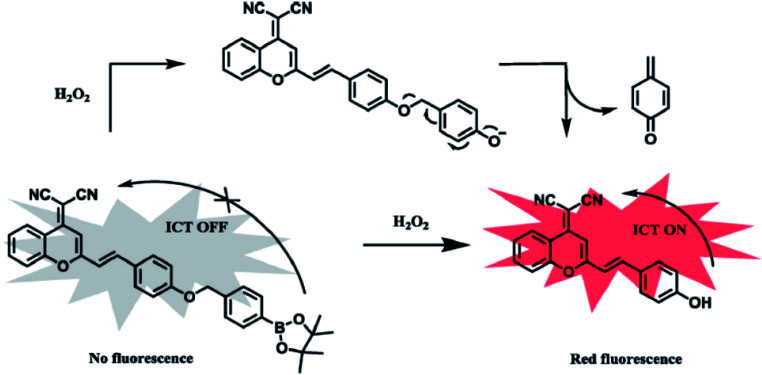
Recognition mechanism of DCM-B toward H_2_O_2_. This figure has been adapted from ref. 42 with permission from Elsevier.

Currently, 2,7-dichlorofluorescin diacetate (DCFH-DA) is the most common and sensitive probe in detecting intracellular ROS.^40^ DCFH-DA itself has no fluorescence, and freely cross the cell membrane. After entering the cell, it will be hydrolyzed into dichlorofluorescin (DCFH) by intracellular esterase. However, DCFH can not go across the cell membranes, making it easy for probes to be loaded into cells. Intracellular ROS (mainly H_2_O_2_) will oxidize the non-fluorescent DCFH into fluorescent DCF (excitation wavelength (*λ*_ex_): 488 nm; emission wavelength (*λ*_em_): 525 nm). So the level of ROS in cells can be indirectly reflected by the intensity of fluorescence. Such reaction is catalyzed by intracellular peroxidase, cytochrome C or Fe(ii). However, DCFH can show cytotoxicity at high concentrations. Also dihydrorhodamines (RhH_2_) can be used to detect H_2_O_2_.^41^ Its detection mechanism is as follows: ROS oxidize RhH_2_ to positive Rh123 with yellow-green fluorescence (*λ*_ex_ = 507 nm; *λ*_em_ = 529 nm), which can penetrate the cell membrane and accumulate under the action of mitochondrial membrane potential, so Rh123 can also be used to detect the mitochondrial membrane potential. It's commonly for detecting H_2_O_2_ by colorimetric method. Recently, Z. Zhou synthetized a colorimetric near-infrared fluorescent probe (*E*)-2-(2-(4-((4-(4,4,5,5-tetramethyl-1,3,2-dioxaborolan-2-yl)benzyl)oxy)styryl)-4*H*-chromen-4-ylidene) malononitrile (DCM-B) to detect H_2_O_2_.^42^ Without H_2_O_2_ in the solution, the intramolecular charge transfer (ICT) process would not occur and no fluorescence could be generated by DCM-B. After the addition of H_2_O_2_, the hydroxyl production restored the fluorescence and thus changed the color (Fig. 6). The concentration of H_2_O_2_ could be judged visually from the color. With the color changing from yellow to purple, the H_2_O_2_ concentration increased. Moreover, both the fluorescence spectra (*λ*_ex_ = 557 nm, *λ*_em_ = 688 nm) and the gradual increase of fluorescence intensity make the detection results convincing.

### 3.2 Detection of O_2_^−^

**Fig. 7:**
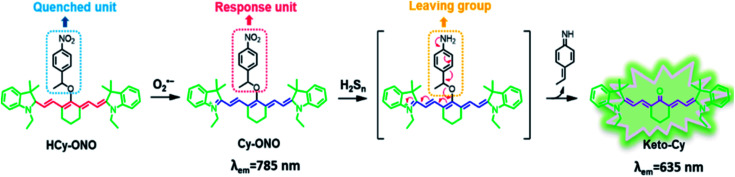
Structure and proposed reaction mechanism of HCy-ONO for H_2_S_*n*_ and O_2_^−^detection. This figure has been adapted from ref. 49 with permission from American Chemical Society.

Dihydroethidine (DHE) can be oxidized to ethidium by O_2_^−^ (*λ*_ex_ = 480 nm, *λ*_em_ = 610 nm) which can be chimeric with DNA and emit red light after aggregation.^43–45^ Alternatively, DHE can react with O_2_^−^ to produce another fluorescent product, which then generates fluorescence while chimeric with DNA.^42,43^ Some of the nanocomposites used in cancer therapy, combined with PDT, can also produce ROS under the irradiation of external light source, so the corresponding probes can be used to detect ROS production. 1,3-Diphenylisobenzofuran (DPBF) can be oxidized by ROS (mainly O_2_^−^) to reduce its UV absorption and fluorescence intensity. Therefore, the change in special UV absorption peak of DPBF before and after the reaction can indirectly reflect the amount of ^1^O_2_ produced by the nanocomposite. Furthermore, DPBF can also detect O_2_^−^ in liposomes.^46^ Same as ROS, reactive sulfur species (RSS) play essential roles in physiological and pathological processes of cells as well. According to reports, hydrogen sulfide (H_2_S, a member of RSS) essentially regulates the intracellular redox status and the fundamental signaling processes.^47,48^ M. Gao *et al.* developed a nitrobenzene derivative functionalized probe HCy-ONO to investigate the cross-talk of H_2_S_*n*_ and O_2_^−^ in living cell and *in vivo*, where the emission wavelengths for O_2_^−^ and H_2_S_*n*_ were 785 nm and 635 nm, respectively. Firstly, the probe HCy-ONO reacted with O_2_^−^ to emit weak fluorescence, and the nitro moiety was thus reduced to an amino group, followed by the 1,6-rearrangement-elimination reaction releasing a cyanine fluorophore with a large Stokes shift (Fig. 7).^49^ The near-infrared region (NIR) fluorescence effectively penetrated tissues, while avoiding the influence of biological auto-fluorescence. The work provides a way to reveal physiological and pathological effects in hypoxic environments.

### 3.3 Detection of ^1^O_2_

**Fig. 8:**
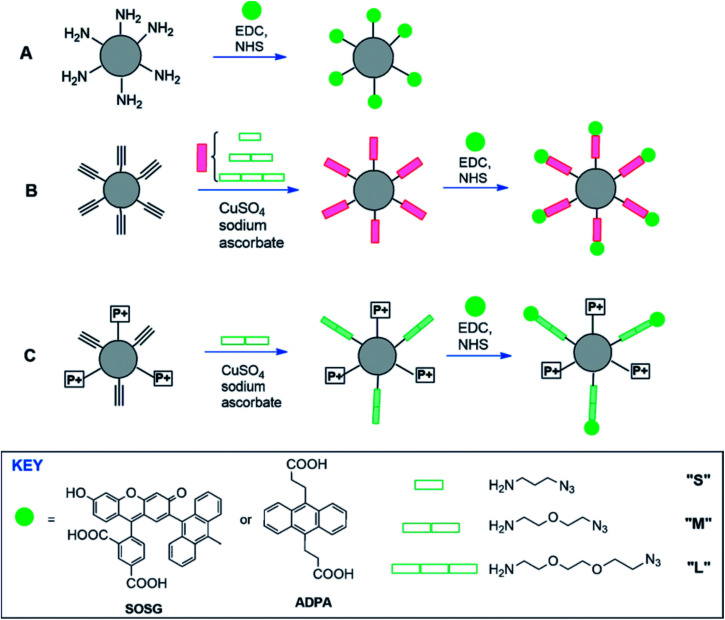
Conjugation of ADPA or SOSG to functionalized polyacryl-amide NPs directly (A) or *via* a spacer (B), and with positively-charged trimethylphosphonium groups (C). EDC: 1-(3-dimethylaminopropyl)-3-ethylcarbodiimide hydrochloride; ADPA: anthracene-9,10-dipropionic acid. This figure has been adapted from ref. 52 with permission from John Wiley and Sons.

^1^O_2_ is the main bactericide of the body's immune system and can also be used for photodynamic therapy of cancer.^50^ Currently, there is a commercially available probe, Singlet Oxygen Sensor Green (SOSG), for the specific detection of singlet oxygen ^1^O_2_, which can be observed even in a short period of time. But, the main drawback of SOSG is that, when irradiated with UV light, its endoperoxide derivative acts as a photosensitizer itself, generating singlet oxygen which then induces more fluorescence emission of SOSG.^51^ SOSG is a typical anthracene ^1^O_2_ based probe which can form stable peroxide by reacting with ^1^O_2_ to block the electron transfer, thereby restoring the intrinsic fluorescence of the fluorescein. As shown in Fig. 8, polyacrylamide nanoparticle as a carrier of SOSG was used to improve the biocompatibility and imaging of SOSG. Amide bonds were formed remotely from the reactive anthracene moiety, which avoided the effect of the microenvironment of SOSG in the nanoprobe on the quenching of electron transfer. The influence of linkers in different lengths (S, M and L) were tested on the performance of the nanoprobe.^52^

Fluorescence probe method is the most common and widely used to detect intracellular ROS, and the fluorescent probes vary with ROS. Generally, the fluorescent probes are basically reductive dyes due to the strong oxidation ability of ROS.^53^ The dye is oxidized to produce fluorescence *via* redox reaction. However, the detection of a certain ROS using a certain fluorescence probe is not accurate since the oxidization of ROS is not significantly different among different fluorescence probes. For example, DCFH-DA could be oxidized not only by H_2_O_2_, but also by OH˙ and ^1^O_2_.^54^ Moreover, some fluorescent probes such as DPBF are highly reductive and easily oxidized even in the air, causing a lot of inconvenience to the experiment. In view of this, although the fluorescent probe method has advantages of simple operation and high sensitivity, the reaction mechanism between fluorescent probes and ROS is unclear due to the short lifetime and high activity of ROS and should be further explored in the future so as to improve the selectivity and the accuracy of fluorescence probe method for ROS detection. Table 1 lists some fluorescent probes previously reported for the detection of H_2_O_2_, O_2_^−^ and ^1^O_2_ and their imaging application, most of which have been mentioned in this article.

**Table 1:** Some fluorescent probes previously reported for the detection of H_2_O_2_, O_2_^−^ and ^1^O_2_ and their imaging application^a^

Probes	*λ*_ex_/*λ*_em_ (nm)	Response time (min)	Imaging application	Ref.
**Detection of H**_**2**_ **O**_**2**_				
(DCFH-DA)	488/525	20	Eukaryotic culture cell	40
RhH_2_	507/529	30	Eukaryotic culture cell	41
(DCM-B)	557/688	30	HepG2 cells	42
FE–H_2_O_2_	360/540	4	HeLa cells	55

**Detection of O**_**2**_^**−**^				
DHE	480/610	At least 10	Living cells	43
DPBF	UV 420	2	Solution	46
HCy-ONO	765/785	3	Mice	49
HCy-SeH	755/800	30	Mice	56

**Detection of** ^**1**^ **O**_**2**_				
SOSG	504/525	1	Solution	51
ASG	350/537	3	Hela cells	57
MTTA-Eu^3+^	294/335	20	Hela cells	58
FN-4	330/378	No data	Solution	59

a DCFH-DA: 2,7-dichlorofluorescin diacetate; RhH_2_: dihydrorhodamines; DCM-B: (*E*)-2-(2-(4-((4-(4,4,5,5-tetramethyl-1,3,2-dioxaborolan-2-yl)benzyl)oxy)styryl)-4*H*-chromen-4-ylidene) malononitrile; FE–H_2_O_2_: H_2_O_2_ reaction site and 4-ferrocenyl(vinyl)pyridine unit; DHE: dihydroethidine; DPBF: 1,3-diphenyl isobenzofuran; SOSG: 2,7-dichlorofluorescin diacetate; ASG: Aarhus Sensor Green; MTTA-Eu^3+^: [4′-(10-methyl-9-anthryl)-2,2′:6′,2′′-terpyridine-6,6′′-diyl]bis(methylenenitrilo) tetrakis-(acetic acid); FN-4: (*E*)-2-(2-(furan-2-yl)vinyl) naphtho[1,2-*d*]oxazole.

## 4. Conclusion

ROS, as a kind of molecules with short lifetime and high oxidative activity, is the product of metabolism of living body. On the one hand, ROS always maintain a certain level of stability in the living body to ensure the normal physiological functions. On the other hand, the metabolic level of tumor cells is much higher than that of normal cells, leading to a higher threshold of oxidation–antioxidation balance and higher oxidative stress within tumor microenvironment. Moreover, once the oxidation–antioxidation balance is broken, cell DNA, lipids and proteins can be damaged, or cell apoptosis and necrosis can be caused. Therefore, it is of great research significance and application prospect to achieve the inhibition and killing of tumor by breaking the ROS balance in tumor microenvironment. In recent years, both the introduction of external materials to produce ROS through PDT and the combination of immunotherapy to affect ROS level have achieved gratifying results for tumor treatment. The mechanism to detect ROS by fluorescence probe is summarized as well. The role of ROS in tumor therapy in the next stage can be considered from two aspects. First, PDT should further improve ROS production under hypoxic and hypoxic conditions in tumor microenvironment and try to start from the aspect of immunotherapy to kill tumor cells by influencing ROS more effectively. Second, the use of fluorescence probe is quite necessary for ROS detection. Efforts should be made as many as possible to improve the specificity and selectivity of each fluorescent probe, and the new fluorescent probe should be further studied to improve their detection efficiency and sensitivity on the premise that ROS has a short lifetime and strong oxidative activity.

Compared with some other ROS-related reviews that mainly specifically focus on nanomedicine,^60^ PDT in cancer therapy^61,62^ and the level of ROS content,^63^ this review is more like a concise outlet to answer the key questions including the sources of ROS, the importance of keeping certain content of ROS in tumor microenvironment and the sketch of the ROS role in cancer therapy. Furthermore, the detection methods for H_2_O_2_, O_2_^−^ and ^1^O_2_ and the corresponding imaging applications are summarized as well. So this review provides a short summarization which helps to quickly and accurately understand the role of ROS in cancer therapy.

## Conflicts of interest

There is no conflict of interest to declare in this manuscript.

## Supplementary Material
